# Integrative Physiological, Transcriptomic, and Functional Analysis Reveals a Positive Contribution of *TaCDPK22-5A* to Drought Adaptation in Wheat

**DOI:** 10.3390/genes17090985

**Published:** 2026-08-22

**Authors:** Bo Liu, Yu Li, Huina Li, Kexin Niu, Hongliang Wang, Luxian Liu

**Affiliations:** 1 School of Agriculture, Henan Institute of Science and Technology, Xinxiang 453003, China; liubo@hist.edu.cn (B.L.); liyu@hist.edu.cn (Y.L.); 18790684054@163.com (H.L.); niukx6@163.com (K.N.); 2 College of Life Sciences, Henan Normal University, Xinxiang 453007, China

**Keywords:** wheat, drought stress, physiological response, transcriptome analysis, CDPK, gene silencing

## Abstract

Background: Drought tolerance in wheat is a complex trait controlled by multiple regulatory networks, among which calcium-dependent protein kinases (CDPKs) act as important components linking stress perception with downstream cellular responses. However, the functional contribution of individual CDPK members to drought adaptation in wheat remains largely unclear. This study aimed to identify and functionally characterize drought-responsive *CDPK* genes associated with differential drought responses in wheat. Methods: Two wheat lines derived from the same breeding background exhibiting contrasting drought adaption, 23B1 and 23B39, were subjected to PEG6000-induced osmotic stress. Growth traits, osmotic adjustment-related metabolites, membrane damage indicators, and antioxidant enzyme activity were evaluated. Transcriptomic analysis was performed at early drought-response stages, followed by differential expression analysis, functional enrichment, CDPK family screening, and qRT-PCR validation. The role of *TaCDPK22-5A* was further investigated using barley stripe mosaic virus (BSMV)-mediated virus-induced gene silencing (VIGS). Results: The drought-responsive line 23B1 maintained stronger growth, accumulated higher levels of proline and soluble sugars, exhibited enhanced peroxidase activity, and showed reduced membrane lipid peroxidation compared with 23B39. Transcriptome analysis revealed extensive transcriptional reprogramming under drought stress, with differentially expressed genes mainly associated with metabolic adjustment, transport regulation, secondary metabolism, and stress-responsive pathways. Among the identified CDPK members, *TaCDPK22-5A* showed a strong drought-responsive expression pattern in the line exhibiting stronger drought tolerance (23B1). Virus-induced gene silencing of *TaCDPK22-5A* significantly impaired drought tolerance, resulting in reduced growth, biomass accumulation, and chlorophyll retention under drought conditions. Conclusions: These findings demonstrate that *TaCDPK22-5A* contributes positively to drought adaptation in wheat and highlight CDPK-mediated calcium signaling as an important regulatory component of drought responses. The identified gene provides a potential target for improving drought resilience in wheat breeding.

## 1. Introduction

Wheat (*Triticum aestivum* L.) is one of the most widely cultivated cereal crops and provides a major source of calories and plant-derived protein for the global population. However, wheat production is increasingly threatened by environmental stresses, among which drought is one of the most severe constraints affecting plant growth, yield stability, and grain quality [1,2]. At the whole-plant level, drought stress restricts cell division and expansion, resulting in reduced shoot growth and plant height. Meanwhile, root system architecture is strongly affected by water limitation. Although moderate drought may promote deeper root development to enhance water acquisition, prolonged or severe drought generally inhibits root elongation and reduces root biomass accumulation [3]. To cope with water deficiency, plants activate a series of physiological adjustments that maintain cellular homeostasis and improve stress adaptation [4,5].

One of the earliest responses to drought stress is excessive production of reactive oxygen species (ROS), which can disrupt cellular structures through oxidative damage and membrane lipid peroxidation. The accumulation of malondialdehyde (MDA), a product of membrane lipid peroxidation, is widely used as an indicator of oxidative injury under drought conditions [6,7]. In contrast, plants enhance drought adaptation through the accumulation of compatible osmolytes, including proline (Pro) and soluble sugars (SSs), which contribute to osmotic adjustment, stabilization of proteins and membranes, and regulation of stress signaling processes [8,9]. Antioxidant enzymes, such as peroxidase (POD), also play important roles in maintaining ROS homeostasis by limiting oxidative damage during dehydration stress. Previous studies have shown that wheat genotypes with improved drought performance generally exhibit higher accumulation of osmoprotective compounds and stronger antioxidant capacity compared with drought-sensitive genotypes [8,10]. Therefore, changes in MDA, Pro, SS, POD activity, root length, and plant height are widely applied as physiological indicators for evaluating drought responses in wheat.

Plant responses to drought are regulated by interconnected signaling networks involving phytohormones, calcium signaling, and transcriptional regulation [11]. Among these pathways, abscisic acid (ABA) signaling represents an important regulatory module during water deficit responses. Drought-induced ABA accumulation is perceived by PYR/PYL/RCAR receptors, which inhibit clade A type 2C protein phosphatases (PP2Cs), thereby releasing SNF1-related protein kinase 2 (SnRK2) activity and activating downstream stress-responsive gene expression [12,13,14]. In parallel, drought stress rapidly induces transient changes in cytosolic calcium concentration, which function as important secondary signals in stress perception [15].

CDPKs are unique calcium sensors in plants because they combine calcium perception and protein kinase activity within a single protein molecule. A typical CDPK contains an N-terminal variable region, a serine/threonine kinase domain, an autoinhibitory junction region, and a calmodulin-like regulatory domain containing four EF-hand calcium-binding motifs. Upon calcium binding, conformational changes relieve autoinhibition and activate kinase activity, allowing CDPKs to directly convert calcium signals into phosphorylation-mediated responses. Increasing evidence indicates that CDPKs participate in diverse drought-responsive processes, including stomatal regulation, osmotic adjustment, ROS metabolism, and ABA-associated stress signaling [16,17]. Genome-wide analyses have identified a large CDPK gene family in wheat, and several members of this family have been implicated in drought adaptation. For example, *TaCDPK1-5A* positively regulates drought tolerance through ABA-dependent stomatal regulation and interactions with mitogen-activated protein kinase signaling pathways [18]. Other members, including *TaCDPK25-U*, *TaCDPK2*, *TaCDPK4*, and *TaCDPK17*, have been linked to alternative splicing regulation, ROS homeostasis, and ABA-mediated stress responses [19,20]. However, the functions of many wheat *CDPK* genes remain unknown, and the contribution of individual CDPK members to drought adaptation requires further investigation.

Sequencing (RNA-seq) provides a comprehensive approach for analyzing genome-wide transcriptional changes and has become an effective strategy for discovering drought-responsive regulatory genes [21,22]. Comparative transcriptome analyses of wheat materials with contrasting drought responses have revealed extensive transcriptional reprogramming associated with ABA signaling, ROS scavenging, carbohydrate metabolism, amino acid metabolism, and secondary metabolite biosynthesis [23]. These studies indicate that drought adaptation is controlled by complex regulatory networks rather than individual pathways or genes. However, transcriptomic data mainly provide associations between gene expression patterns and stress responses, and functional validation is required to determine whether candidate genes directly contribute to drought adaptation [24].

Drought response in wheat is influenced by multiple genetic factors. Transcriptional differences identified between wheat materials may reflect both stress-responsive regulation and genetic background variation. Therefore, integrating comparative transcriptomics with functional characterization provides a more reliable strategy for identifying biologically relevant drought regulators. In this study, two wheat lines sharing the common founder parent “Zhou8425B” exhibiting contrasting drought responses were used as a comparative system to investigate physiological and molecular changes associated with drought adaptation. Through physiological characterization, transcriptome profiling, CDPK family screening, qRT-PCR validation, and BSMV-mediated virus-induced gene silencing (VIGS), we identified and functionally characterized the drought-responsive CDPK gene *TaCDPK22-5A*. Our findings provide new insights into calcium-associated regulation of drought responses and identify a potential candidate gene for improving drought resilience in wheat breeding programs.

## 2. Materials and Methods

### 2.1. Plant Materials and Simulated Drought Treatment

Two independently constructed wheat lines, 23B1 and 23B39, were selected, which share the common founder parent “Zhou8425B”. 23B1 mainly consists of Huanghuai winter wheat germplasm incorporated with an in-house mutant line 03011, while 23B39 integrates germplasm from the Huanghuai wheat region and the Yangmai-related genetic components. Healthy, uniform seeds were selected and rinsed twice in distilled water for 10–15 min each time to remove surface impurities. Seeds were then placed in 9 cm Petri dishes lined with filter paper for germination, with 30 seeds per dish and three replicate dishes per line. Seeds were oriented with the embryo facing down, and Petri dishes were incubated in a growth chamber at 16 °C under a 16 h (light)/8 h (dark) photoperiod with a light intensity of approximately 22,000 lux. During the first 3 days, distilled water was added daily to maintain sufficient moisture to facilitate uniform germination.

After three days of germination, seedlings with similar growth status were transferred to hydroponic culture containing 1/4 MS medium (M524, PhytoTechnology Co., Ltd., Beijing, China), supplemented with 0% (Control), 10%, or 20% (*w*/*v*) polyethylene glycol 6000 (PEG6000, P8250, Solarbio Technology Co., Ltd., Beijing, China) to simulate drought stress [25,26]. Each hydroponic container held 100 mL of the treatment medium, and the solutions were renewed every 3 days. Seedlings were cultured for 6 days in the same growth chamber conditions described above. On day 9, shoot or root length was measured with a ruler from root–shoot junction to shoot apex or root tip; fresh weight (FW) was measured immediately after treatment and removing surface moisture; dry weight (DW) was measured after oven-drying at 65 °C until reaching a constant weight; and physiological indicators of seedlings were measured used the methods in Section 2.2. For transcriptomic analysis, the seedlings were germinated for three days before PEG treatment. On day 3, 10% PEG6000 treatment was initiated at 6 h and 12 h time points to ensure consistent growth time among sampled materials. The 0 h (Control, also as CK), 6 h and 12 h samples were collected at the same time after the initiation of PEG treatment. Leaf samples were immediately frozen in liquid nitrogen and stored at −80 °C until further use.

### 2.2. Measurement of Physiological Indicators

#### 2.2.1. Malondialdehyde (MDA) Assay

MDA was measured using the MDA Assay Kit (G0109W, Suzhou Comin Biotechnology Co., Ltd., Suzhou, China) [27,28]. Leaf tissue (~0.01 g) was homogenized in 200 μL of extraction buffer. The homogenate was kept on ice and centrifuged at 12,000 rpm for 10 min at 4 °C. The supernatant (90 μL) was mixed with 135 μL of reagent I from the kit, incubated in a 95 °C water bath for 30 min to allow reaction with thiobarbituric acid, and immediately cooled on ice. Samples were centrifuged at 10,000 rpm for 10 min at 25 °C, and 30 μL of the resulting supernatant was transferred to a 96-well microplate. Absorbance was measured at 532 nm and 600 nm, and MDA content (nmol/g fresh weight) was calculated using the formula provided by the manufacturer. Three biological replicates were performed per sample.

#### 2.2.2. Proline (Pro) Assay

Pro was measured using the Proline Assay Kit (G0111W, Suzhou Comin Biotechnology Co., Ltd., Suzhou, China) [29]. Leaf tissue (~0.02 g) was homogenized in 400 μL of extraction buffer and extracted by water bath at 90 °C for 10 min. The mixture was centrifuged at 12,000 rpm for 10 min at 25 °C. The supernatant (112.5 μL) was mixed with 112.5 μL of ice-cold acetic acid and 225 μL of reagent I, incubated at 95 °C for 30 min, cooled, and 50 μL was transferred to a 96-well plate. Absorbance at 520 nm was measured, and proline concentration (μg/g fresh weight) was determined using a standard curve.

#### 2.2.3. Soluble Sugar (SS) Assay

Soluble sugars were quantified using the Soluble Sugar Assay Kit (G0501W, Suzhou Comin Biotechnology Co., Ltd., Suzhou, China) [30]. Leaf tissue (~0.05 g) was homogenized in 150 μL of 80% ethanol, extracted at 50 °C for 20 min, and centrifuged at 12,000 rpm for 10 min at room temperature. The supernatant (15 μL) was mixed with 45 μL of distilled water, 18 μL of working reagent, and 150 μL concentrated sulfuric acid, incubated at 95 °C for 10 min, cooled, and 60 μL was transferred to a 96-well plate. Absorbance at 620 nm was measured, and SS content (μg/g fresh weight) was calculated using a standard glucose curve. Three biological replicates were analyzed per sample.

#### 2.2.4. Peroxidase (POD) Activity Assay

POD activity was quantified using the Plant Peroxidase activity Assay Kit (G0107W, Suzhou Comin Biotechnology Co., Ltd., Suzhou, China) [30]. Leaf tissue (~0.1 g) was homogenized in extraction buffer on ice and centrifuged at 12,000 rpm for 10 min at 4 °C. The supernatant (10 μL) was transferred to a 96-well microplate and then mixed with 40 μL of reagent I, 140 μL of reagent II, and 10 μL of reagent III from the kit. Absorbance was measured at 470 nm, and POD activity (U/g fresh weight) was calculated using the formula provided by the manufacturer. Three biological replicates were performed per sample.

#### 2.2.5. Chlorophyll Content Assay

Leaf chlorophyll relative content was determined using a portable SPAD chlorophyll meter (SPAD-502plus, Konica Minolta, Tokyo, Japan). Uniform and fully expanded functional leaves of wheat were selected for measurement at the designated growth stage. After calibrating the instrument, three detection points were selected on each leaf blade avoiding leaf veins and leaf margins. The SPAD values were recorded, and the average value of each leaf was calculated. Three biological replicates were performed.

### 2.3. RNA Extraction and cDNA Obtained

Total RNA was extracted from approximately 100 mg of leaf tissue per sample using the Total RNA Simple Extraction Kit (DP419, Tiangen Biotech Co., Ltd., Beijing, China). RNA quality and concentration were assessed by agarose gel electrophoresis and spectrophotometry. High-quality RNA (≥1 μg) was used to construct strand-specific cDNA libraries with the NEBNext^®^ Ultra™ II RNA Library Prep Kit for Illumina and NEBNext^®^ Ultra™ Directional RNA Library Prep Kit for Illumina (E7770/7760, New England Biolabs Inc., Ipswich, MA, USA). Poly (A)^+^ mRNA was enriched using oligo (dT) magnetic beads and fragmented under divalent cation conditions. First-strand cDNA was synthesized using random hexamer primers, followed by second-strand synthesis. Double-stranded cDNA was purified, end-repaired, A-tailed, and ligated to sequencing adapters. Fragments of 400~500 bp were selected with AMPure XP beads, PCR-amplified, and purified to generate high-quality libraries for sequencing.

### 2.4. RNA-Seq Data Analysis

Raw reads were processed using fastp v0.22.0 to remove adapters and low-quality reads (average quality score < Q20) [31]. Clean reads were aligned to the wheat reference genome (IWGSC RefSeq v2.1) using HISAT2 v2.1.0 [32]. Gene counts were obtained with HTSeq v0.9.1 and normalized as FPKM. Differentially expressed genes (DEGs) were identified using DESeq2 v1.38.3, with |log_2_FoldChange| > 1 and adjusted *p*-value < 0.05 considered significant [33]. GO and KEGG enrichment analyses were performed with clusterProfiler v4.6.0 (*p* < 0.05) and TBtools v2.362 [34]. The annotation of DEGs was analyzed by Cloud Analysis tools provided by Personal (Shanghai Personal Biotechnology Co., Ltd., Shanghai, China) at https://www.genescloud.cn/cloudToolProcess/overview (accessed on 10 November 2025) based on the Swiss-Prot database.

### 2.5. Virus-Induced Gene Silencing (VIGS)

#### 2.5.1. Vector Construction

Specific VIGS fragments targeting *TaCDPK22-5A* were selected using the SGN-VIGS tool at https://vigs.solgenomics.net/ (accessed on 13 December 2025) to assess sequence similarity and the potential for co-silencing. Primers with BSMV-γ vector adaptors (*TaCDPK22-5A*-VIGS-F/R) were used to amplify these fragments. Recombinant plasmids (BSMV-γ::*TaCDPK22-5A*), alongside control plasmids including wild-type BSMV-α, BSMV-β, BSMV-γ, and the positive control BSMV-γ-*PDS*-as (targeting phytoene desaturase), were extracted using plasmid miniprep kit. For vector linearization, BSMV-β was digested with Spe I, whereas BSMV-α, BSMV-γ, BSMV-γ-*PDS*-as, and recombinant BSMV-γ plasmids were digested with Mlu I.

#### 2.5.2. In Vitro Transcription and Viral RNA Inoculation

The linearized DNA templates were transcribed in vitro to produce capped viral RNA using the RiboMAX™ Large Scale RNA Production System-T7 (Promega, Madison, WI, USA) under strict RNase-free conditions. Wheat seedlings at the two-leaf stage were used for inoculation. The viral inoculum was prepared by mixing 3 μL each of BSMV-α, BSMV-β, and corresponding BSMV-γ (or recombinant γ) transcripts. The 9 μL mixture was diluted with an equal volume of DEPC-treated water and thoroughly homogenized with 350 μL FES buffer via gentle pipetting. For each plant, 9.5 μL of the mixture was applied to a gloved finger and gently rubbed onto the second wheat leaf about 10 times. Inoculated leaves were immediately sprayed with a fine mist of sterile DEPC-treated water to prevent drying. Plants were kept in the dark under a black hood for 24 h to maintain high humidity, then moved to a growth room set at 22 °C with a 16 h light/8 h dark cycle. Each group had three biological replicates (3 pots per treatment). Around 10 days post-inoculation, when BSMV-γ-*PDS*-as plants showed photobleaching, leaves were harvested to measure silencing efficiency by RT-qPCR. To test drought response, another set of silenced plants from the same batch was treated with 10% (*w*/*v*) PEG6000.

### 2.6. Quantitative Real-Time PCR Validation

Gene specific primers were designed (Supplementary Table S1) using Primer Premier 5.0 and validated via NCBI Web BLAST at https://blast.ncbi.nlm.nih.gov/ (accessed on 3 February 2026) against the NCBI database. For genes with closely related homoeologous copies, primer sequences were designed based on the corresponding reference sequences and checked in silico against the A-, B-, and D-genome homoeologs to minimize potential non-specific amplification. The primer sequences and their corresponding target genes are listed in Supplementary Table S1. First-strand cDNA was synthesized from total RNA using the FastKing RT Kit (with gDNase) from Tiangen Biotech Co., Ltd., Beijing, China. qRT-PCR was conducted using ChamQ SYBR qPCR Master Mix (Vazyme Biotech Co., Ltd., Nanjing, China) on a LightCycler^®^ 96 Real-Time PCR System (Roche, Basel, Switzerland). Each reaction included three biological and three technical replicates, with wheat actin as the internal control. Relative expression was calculated using the 2^−ΔΔCt^ method [35]. Genes were prioritized for qRT-PCR based on significant differential expression, absolute log2 fold change (>1), adjusted *p*-value (<0.05), and genotype-dependent expression patterns.

### 2.7. Statistical Analysis

Data are presented as means ± standard deviation (SD) from independent biological replicates. For physiological measurements involving genotype and PEG treatment, two-way analysis of variance (ANOVA) was used to evaluate the effects of genotype, PEG concentration, and their interaction. When significant effects were detected, multiple comparisons were performed using an appropriate post hoc test. For time-course expression analyses, the effects of genotype and treatment/time were evaluated according to the experimental design. For pairwise comparisons, multiple unpaired *t* tests were used where a predefined two-group comparison was appropriate (* represents *p* < 0.05, ** represents *p* < 0.01, *** represents *p* < 0.001, **** represents *p* < 0.0001; ns represents not significant).

## 3. Results

### 3.1. Effects of PEG-Induced Drought Stress on Wheat Seedling Growth

**Figure 1:**
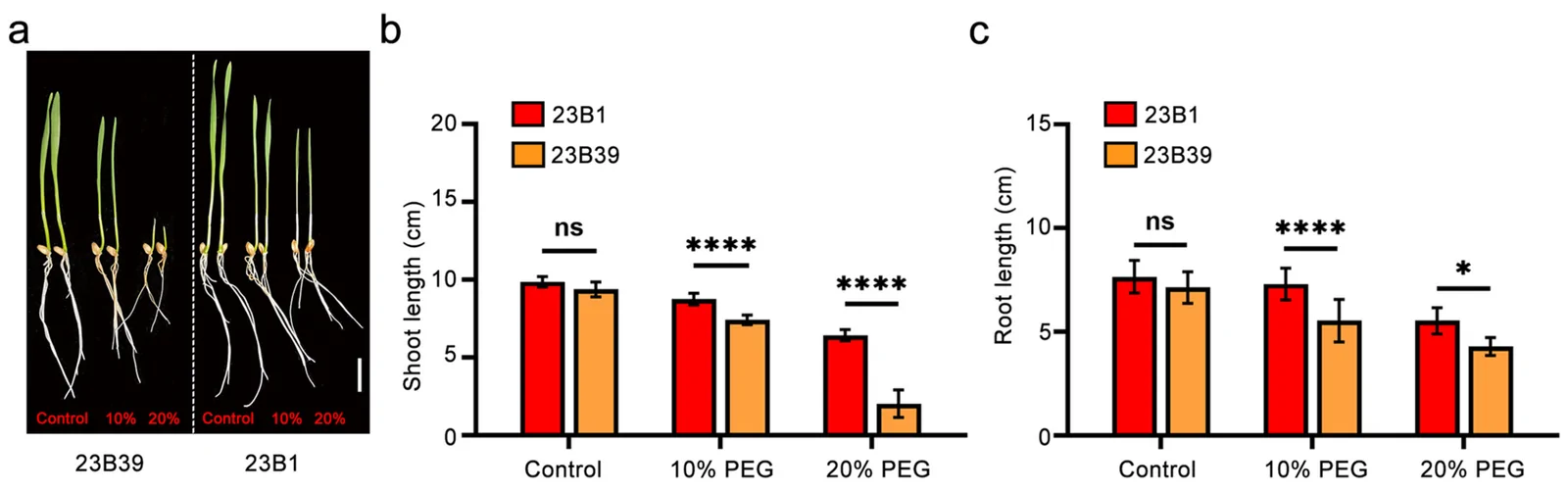
Effects of drought stress on physiological traits of wheat lines 23B1 and 23B39. (**a**) Morphological changes in 23B1 and 23B39 under drought stress. Control represents no PEG6000 treatment, 10% and 20% represent treatment with 10% or 20% PEG6000 to simulate the drought stress, scale bar = 1 cm. (**b**,**c**) Shoot and root length of wheat lines 23B1 and 23B39 under drought stress. Error bars represent the standard deviation (SD) of three biological replicates. Significant differences were determined using two-way ANOVA (* *p* < 0.05, **** *p* < 0.0001; ns, not significant).

To characterize the drought responses of wheat lines 23B1 and 23B39, seedlings were grown in liquid MS medium supplemented with 10% or 20% (*w*/*v*) PEG6000 for six days to impose osmotic stress. PEG treatment resulted in significant growth inhibition in both lines, and the inhibitory effects increased with increasing PEG concentration. However, the extent of growth reduction differed between the two lines. Compared with 23B39, 23B1 maintained relatively stronger shoot growth and root development under PEG-induced stress conditions (Figure 1a). Following 10% PEG treatment, shoot elongation was reduced in both lines, but the reduction was less pronounced in 23B1. Under 20% PEG treatment, shoot growth was severely inhibited in both lines, particularly in 23B39, which showed a reduction from approximately 10 cm to 2 cm, whereas 23B1 retained a significantly greater shoot length (approximately 6.5 cm) (*p* < 0.0001) (Figure 1b). Root growth showed a similar response pattern (Figure 1c). No significant difference in root length was observed between the two lines under non-stress conditions. However, PEG treatment markedly inhibited root elongation, with 23B1 maintaining longer roots than 23B39 under both 10% PEG (*p* < 0.0001) and 20% PEG conditions (*p* < 0.05). These results demonstrate that the two wheat lines exhibited distinct growth responses to osmotic stress, with 23B1 showing a greater capacity to maintain shoot and root development under PEG-induced water limitation.

### 3.2. Physiological Response of 23B1 and 23B39 Under Drought Stress

**Figure 2:**
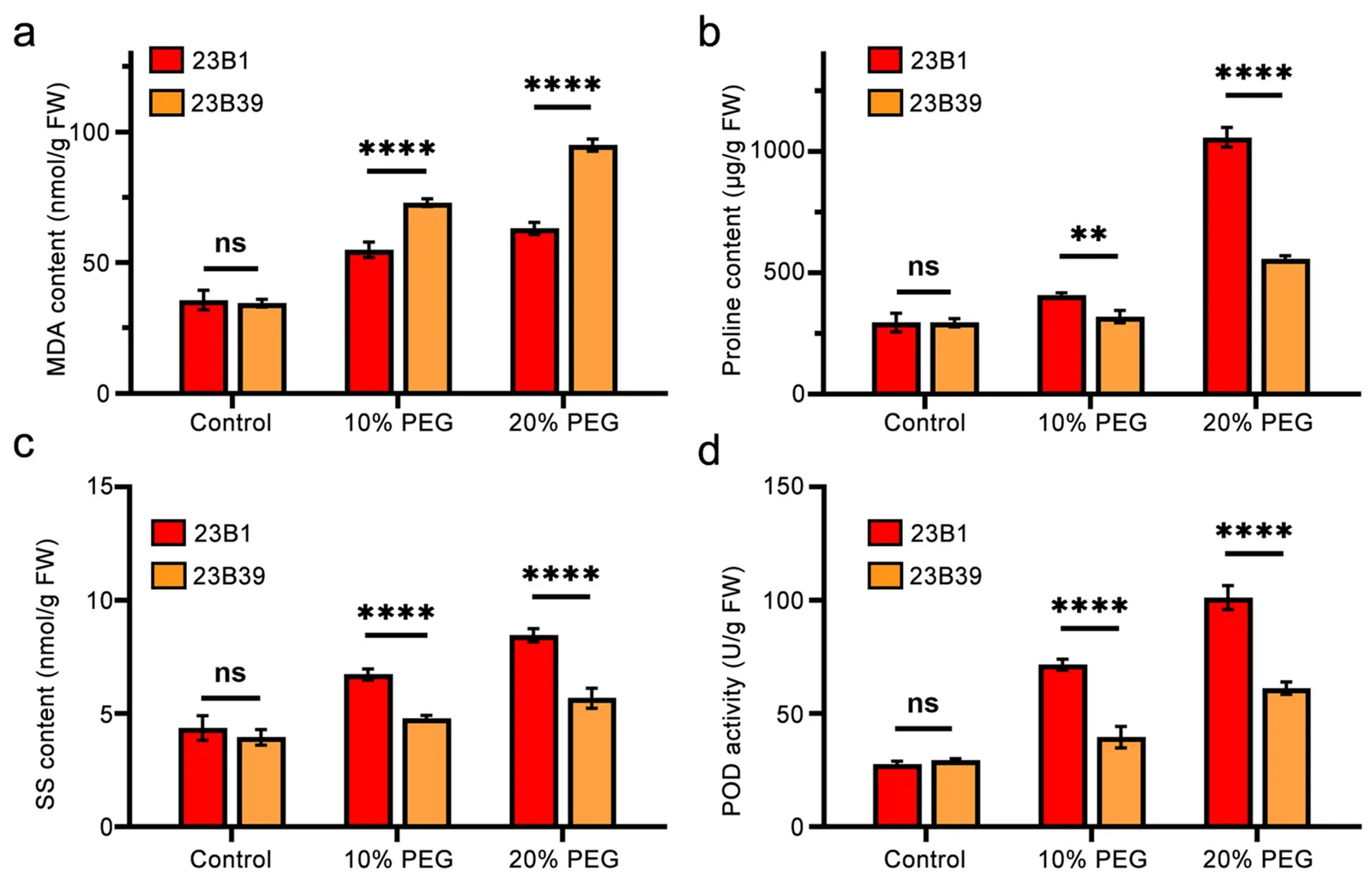
Comparison of physiological parameters in wheat lines 23B1 and 23B39 under control and drought treatments, including (**a**) MDA content; (**b**) Pro content; (**c**) SS content; and (**d**) POD activity. Error bars represent the standard deviation (SD) of three biological replicates. Significant differences were determined using two-way ANOVA (** *p* < 0.01, **** *p* < 0.0001; ns, not significant).

To further characterize the physiological differences associated with contrasting drought responses between the two wheat lines, malondialdehyde (MDA), proline (Pro), soluble sugar (SS), and peroxidase (POD) activity were measured following treatment with 10% and 20% PEG6000 (Figure 2). PEG-induced drought stress resulted in increased MDA accumulation in both lines; however, the increase was more pronounced in 23B39 than in 23B1 under both stress conditions (Figure 2a), indicating a higher level of membrane lipid peroxidation in 23B39.

In contrast, 23B1 exhibited stronger accumulation of osmoprotective compounds under drought conditions. Proline content increased significantly in both lines after PEG treatment, but reached higher levels in 23B1, particularly under 20% PEG treatment, where the content was approximately two-fold higher than that in 23B39 (Figure 2b). Similarly, soluble sugar content showed a greater increase in 23B1 than in 23B39 under both PEG concentrations (Figure 2c).

POD activity was also enhanced by drought stress in both wheat lines, with 23B1 maintaining significantly higher activity than 23B39 under both 10% and 20% PEG treatments (Figure 2d). The factorial analysis testing genotype × treatment using two-way ANOVA shows that the two lines respond significantly differently to stress. Together, these results indicate that the two wheat lines displayed distinct physiological responses to PEG-induced drought stress. Compared with 23B39, 23B1 showed stronger osmotic adjustment capacity, enhanced antioxidant activity, and reduced membrane oxidative damage under water-deficit conditions.

These physiological differences prompted further investigation of the transcriptional regulatory networks underlying the contrasting drought responses of the two wheat lines.

### 3.3. Comparative Transcriptome Profiling of 23B1 and 23B39 Under Drought Stress

**Figure 3:**
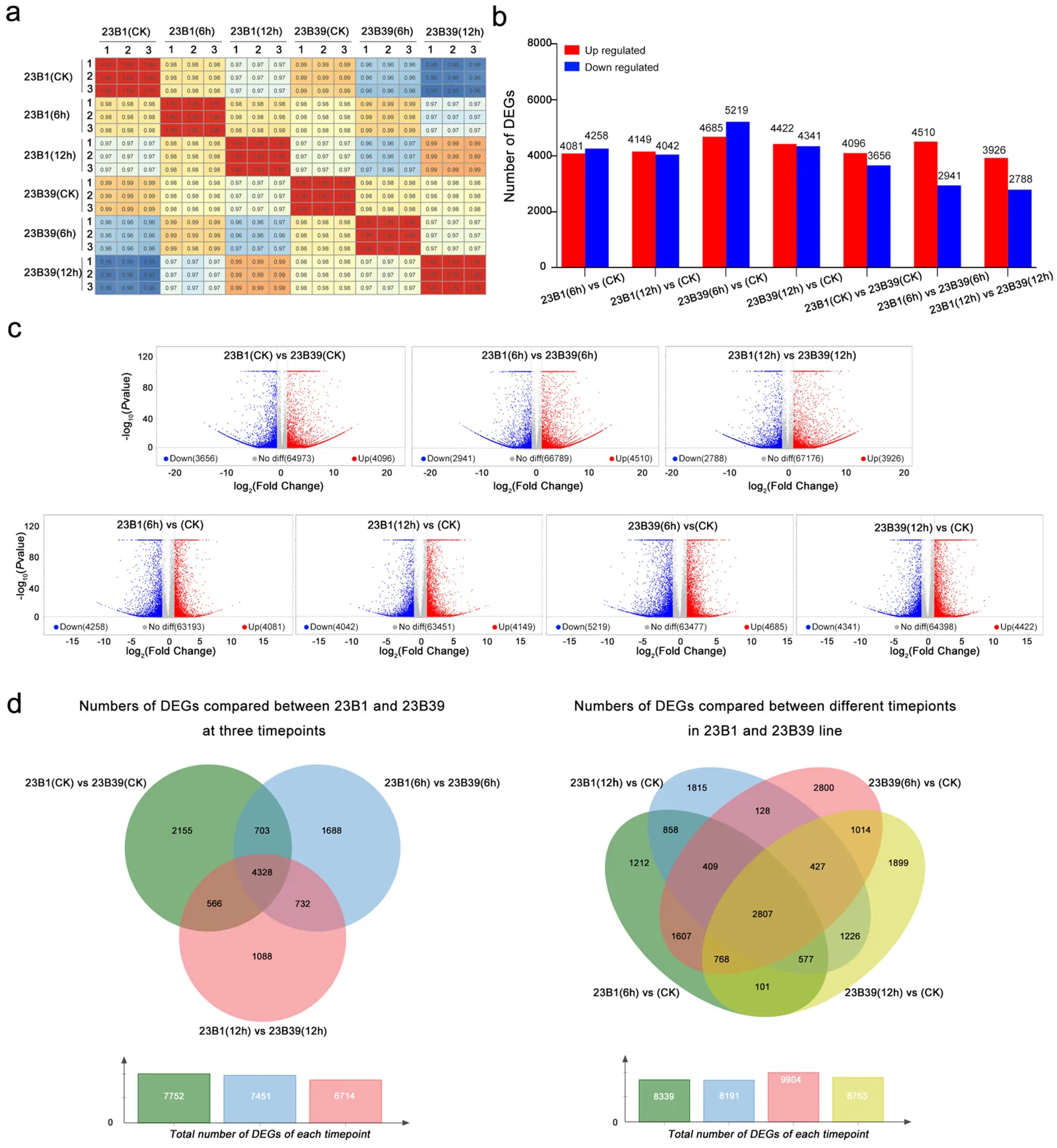
Comparative transcriptome analysis of wheat lines 23B1 and 23B39 under drought stress. (**a**) Pearson correlation analysis showing the correlation among three biological replicates of 23B1 and 23B39 under control (CK), 6 h, and 12 h PEG6000 treatments. (**b**) Numbers of upregulated and downregulated differentially expressed genes (DEGs) identified from pairwise comparisons between the two wheat lines and among different PEG treatment conditions. Red and blue bars indicate upregulated and downregulated DEGs, respectively. (**c**) Volcano plots showing the distribution of DEGs identified from comparisons between 23B1 and 23B39 at the same treatment time points and between PEG-treated samples and their corresponding controls. Red, blue, and gray dots represent upregulated, downregulated, and non-significantly changed genes, respectively. (**d**) Venn diagrams showing the numbers of shared and genotype- or treatment-specific DEGs among different comparison groups. Green, blue, and red circles or columns below represent the number size of different groups.

To investigate the transcriptional changes associated with contrasting drought responses between 23B1 and 23B39, leaf samples were collected at 0, 6, and 12 h after treatment with 10% PEG6000. Three biological replicates were prepared for each treatment, generating a total of 18 RNA-seq libraries. Transcriptome sequencing generated 229.12 Gb of clean data, with more than 9.65 Gb obtained from each library. All libraries showed high sequencing quality, with Q20 values above 99%, Q30 values above 95%, and GC contents ranging from 54.03% to 55.9% (Supplementary Table S2). More than 96% of clean reads were successfully mapped to the wheat reference genome, with approximately 92% uniquely aligned reads, confirming the reliability of the sequencing dataset. The reproducibility of the RNA-seq data was evaluated using Pearson correlation analysis among biological replicates. As shown in Figure 3a, biological replicates within each treatment group exhibited strong correlations (r > 0.95), indicating high consistency among samples and supporting subsequent transcriptomic analyses.

Differentially expressed genes (DEGs) were identified based on comparisons between 23B1 and 23B39 under control and PEG-treated conditions. Under control conditions, 7752 DEGs were detected between the two lines, including 4096 upregulated and 3656 downregulated genes in 23B1 relative to 23B39. Following 6 h PEG treatment, 7451 DEGs were identified between the two lines, indicating that drought stress further altered transcriptional differences between the two genotypes. Volcano plots illustrated the distribution of differentially expressed genes under different comparisons (Figure 3b), showing extensive transcriptional divergence between 23B1 and 23B39 under both normal and drought conditions.

Venn diagram analysis further identified shared and genotype-specific DEGs among different comparisons (Figure 3c). A total of 7752, 7451, and 6714 specific DEGs were detected between 23B1 and 23B39 under control, 6 h PEG, and 12 h PEG conditions, respectively, whereas 4328 genes were shared among all three comparisons. In addition, transcriptome comparison within each line revealed extensive drought-responsive transcriptional changes. In 23B1, 8339 and 8191 DEGs were identified after 6 h and 12 h PEG treatment, respectively, while 23B39 showed 9904 and 8763 DEGs at the corresponding time points. Among these drought-responsive genes, 2807 DEGs were commonly regulated in both lines.

Overall, these results demonstrate that PEG-induced drought stress triggered extensive transcriptional reprogramming in both wheat lines, while the distinct expression profiles between 23B1 and 23B39 provide potential candidates for understanding the molecular basis underlying their contrasting drought responses.

### 3.4. Comparative Transcriptome Analysis Reveals Distinct Drought-Responsive Transcriptional Programs Between 23B1 and 23B39

**Figure 4:**
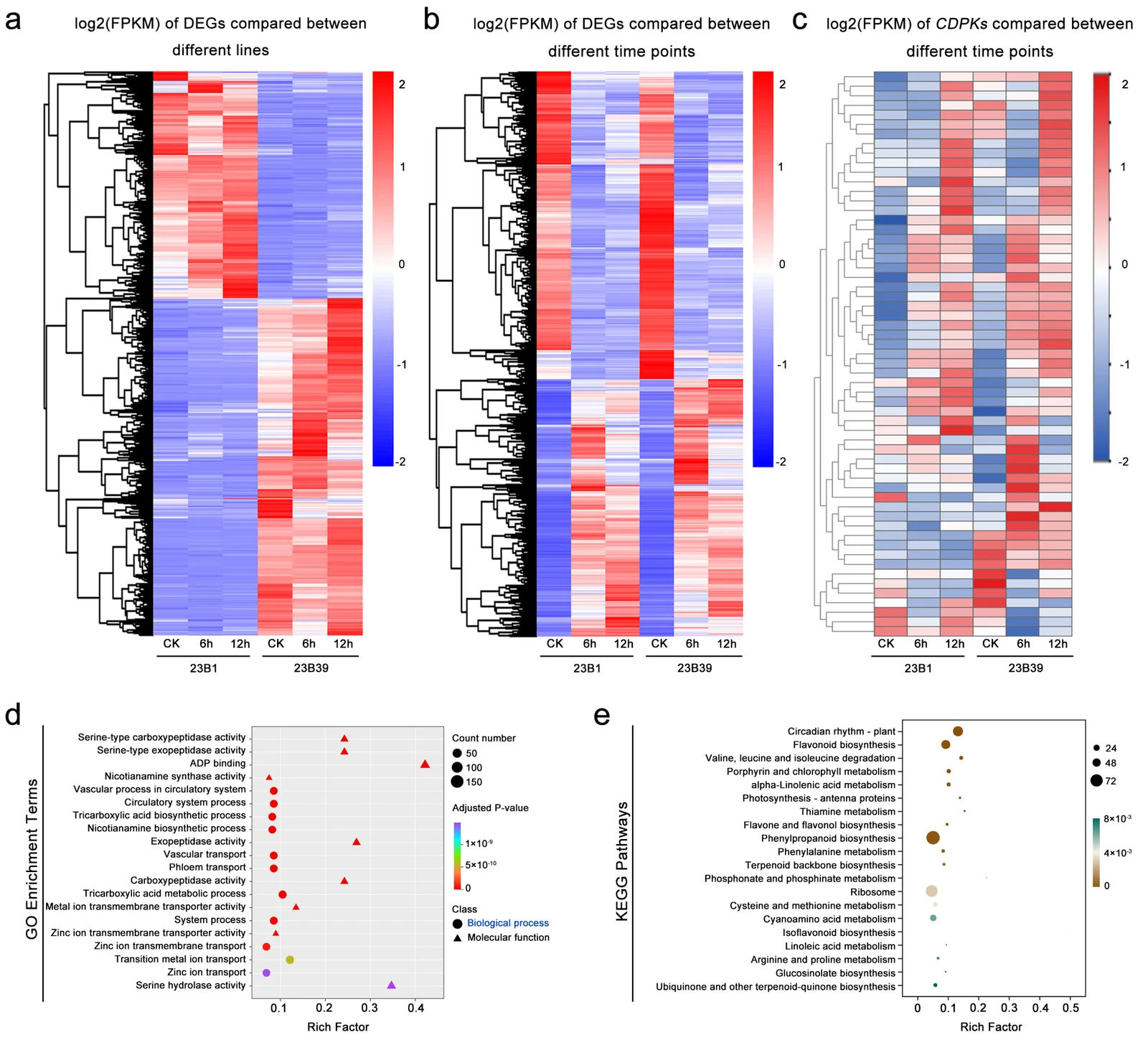
Expression profiles of DEGs, drought-responsive *CDPK* genes and functional enrichment analysis identified from transcriptome analysis under drought stress. Hierarchical clustering heatmaps showing the expression patterns of DEGs compared between 23B1 and 23B39 in each time point (**a**), DEGs identified between different time points in each wheat line after PEG treatments (**b**), and drought-responsive CDPK-like candidates between different time points in each wheat line (**c**). Gene expression levels were calculated based on FPKM values and normalized as log2(FPKM). Red and blue indicate relatively high and low expression levels, respectively. (**d**) Gene Ontology (GO) enrichment analysis of drought-responsive DEGs identified from the 10% PEG6000 versus control comparisons within 23B1 and 23B39. The enriched terms are classified according to biological process and molecular function categories. (**e**) Kyoto Encyclopedia of Genes and Genomes (KEGG) pathway enrichment analysis of drought-responsive DEGs identified from the 10% PEG6000 versus control comparisons within 23B1 and 23B39. The dot size indicates the number of genes involved in each pathway, and the color represents the enrichment significance (Adjusted *p*-value).

To investigate the transcriptional basis underlying the contrasting drought responses of the two wheat lines, differentially expressed genes (DEGs) were identified using DESeq2 with the criteria of |log2FoldChange| > 1 and adjusted *p*-value (padj) < 0.05. Hierarchical clustering analysis showed clear separation of transcriptomic profiles between 23B1 and 23B39 under both control and PEG-treated conditions (Figure 4a,b). The two genotypes displayed distinct expression patterns before drought treatment, indicating genotype-dependent differences in basal transcriptional regulation. Following PEG treatment, both lines exhibited substantial transcriptional reprogramming, with progressive changes observed between 6 h and 12 h after stress exposure. Samples from the same genotype and treatment were clustered together, demonstrating high consistency among biological replicates and confirming the reliability of the transcriptome datasets.

To further characterize drought-responsive pathways, DEGs from the comparisons between PEG-treated and corresponding control within each line were subjected to GO and KEGG enrichment analyses (Figure 4d,e). In both wheat lines, drought-responsive genes were significantly enriched in biological processes associated with transport regulation, tricarboxylic acid metabolism, nicotianamine biosynthesis, and metal ion homeostasis. Molecular function enrichment revealed the involvement of transporter activity, serine-type carboxypeptidase activity, and nucleotide-binding-related functions. KEGG analysis further demonstrated that drought-responsive genes were mainly associated with metabolic pathways, including flavonoid biosynthesis, phenylpropanoid biosynthesis, amino acid metabolism, photosynthesis-related pathways, and secondary metabolite biosynthesis. These results indicate that PEG-induced drought stress triggers extensive transcriptional adjustments involving metabolism, transport, and cellular homeostasis in both wheat lines. Although both genotypes activated common drought-responsive pathways, the different expression patterns between 23B1 and 23B39 suggested that distinct regulatory components may contribute to their contrasting drought adaptation. Among the multiple regulatory pathways affected by drought stress, calcium-mediated signaling represents one of the earliest and most conserved mechanisms involved in stress perception and signal transduction. Changes in intracellular Ca^2+^ concentration serve as important secondary signals that activate downstream protein kinases and transcriptional regulators. Therefore, genes associated with calcium signaling may represent key components contributing to the differential drought responses observed between 23B1 and 23B39. To investigate the potential involvement of calcium-dependent protein kinase (CDPK/CPK)-mediated signaling in the transcriptional response to PEG treatment, we screened the DEG dataset for genes annotated as CDPK/CPK-related genes based on the Swiss-prot Blast.

A total of 78 CDPK-related DEGs across the examined comparisons were identified, and their expression patterns were extracted for comparative analysis (Figure 4c and Supplementary Table S3). Several *CDPK* genes showed pronounced genotype-dependent expression patterns between 23B1 and 23B39. These genotype-dependent CDPK-related DEGs expression patterns suggested that specific members of the CDPK family may contribute to differential drought adaptation and provided candidates for subsequent validation.

### 3.5. qRT-PCR Analysis of Candidate CDPK Genes Under Drought Stress

**Figure 5:**
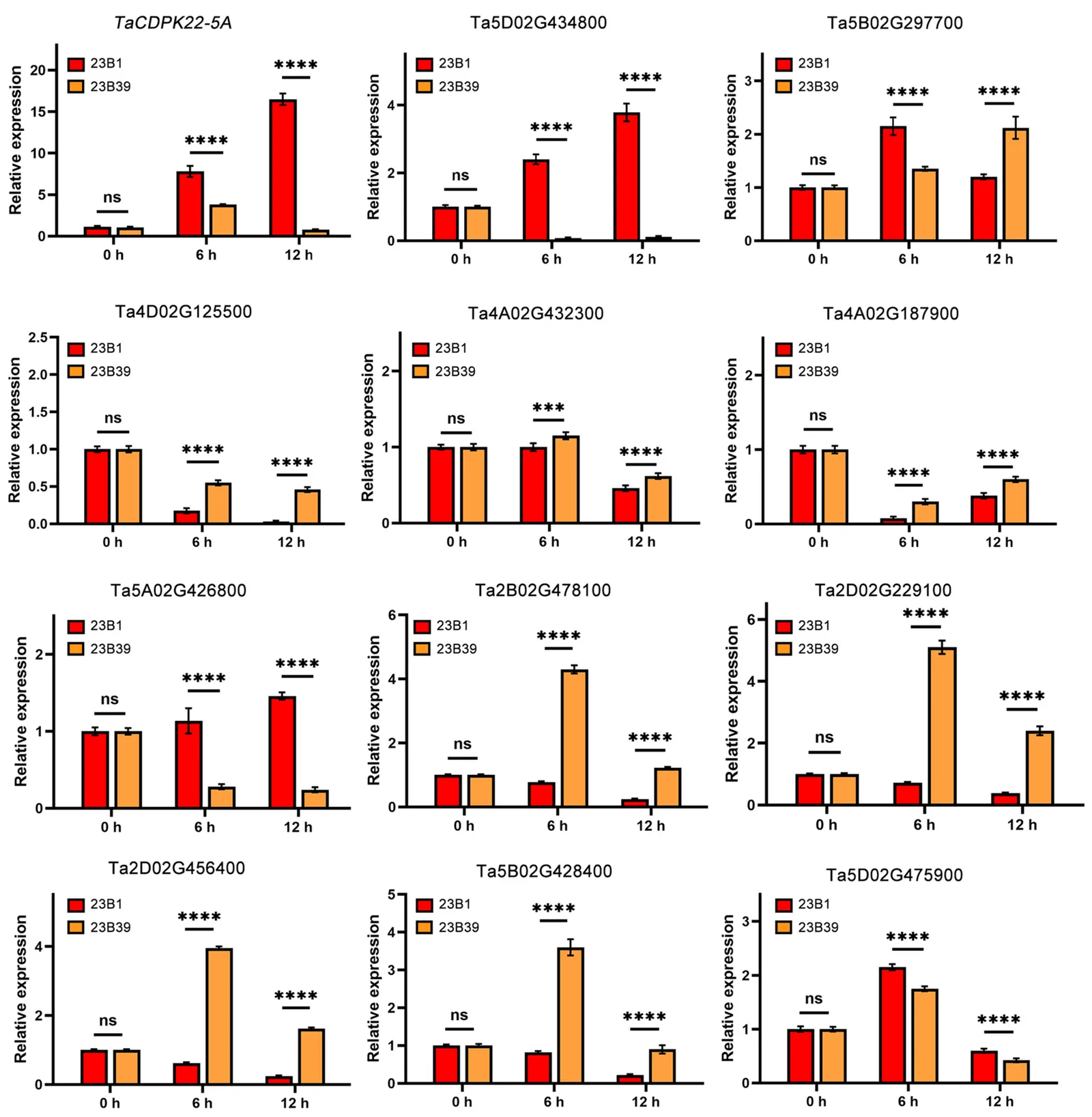
Expression analysis of selected CDPK-related DEGs in wheat lines 23B1 and 23B39 under drought stress. Relative expression levels of candidate *CDPK* genes were determined by quantitative real-time PCR (qRT-PCR) after PEG treatment for 0 h, 6 h, and 12 h. Red and orange bars represent 23B1 and 23B39, respectively. Error bars represent the standard deviation (SD) of three biological replicates. Significant differences were determined using multiple unpaired *t* tests *** *p* < 0.001, **** *p* < 0.0001; ns, not significant).

Based on the transcriptome analysis of 23B1 and 23B39, the representative twelve members of 78 CDPK-related DEGs based on significant differential expression and genotype-dependent expression patterns analyzed by RNA-seq were selected to analyze genotype- and time-dependent expression patterns. *TaCDPK22-5A*, Ta5D02G434800, Ta5A02G426800, and Ta5D02G475900, were preferentially induced in the drought-tolerant line 23B1 (Figure 5). Among these genes, *TaCDPK22-5A* and Ta5D02G434800 showed the strongest responses, with transcript levels increasing continuously during the treatment and reaching the highest levels at 12 h, whereas only weak or negligible induction was detected in 23B39. In contrast, Ta2B02G478100, Ta2D02G229100, Ta2D02G456400, and Ta5B02G428400 were markedly upregulated in 23B39, but remained low or declined in 23B1. Ta5B02G297700 was induced in both lines, although the timing and magnitude of induction differed, while Ta4D02G125500, Ta4A02G432300, and Ta4A02G187900 were generally downregulated during drought treatment. Notably, Ta4D02G125500 declined more rapidly in 23B1, whereas the reduction was less pronounced in 23B39. Overall, the qRT-PCR results revealed substantial differences in the regulation of CDPKs between the two wheat lines. *TaCDPK22-5A* was selected for further analysis of its biological function in drought response because its high induction (~8-fold at 6 h and ~18-fold at 12 h in 23B1) was consistent across RNA-seq and qRT-PCR and showed a clear genotype-dependent response, making it a priority candidate for functional testing.

### 3.6. Functional Assessment of TaCDPK22-5A in Response to PEG-Induced Osmotic Stress

**Figure 6:**
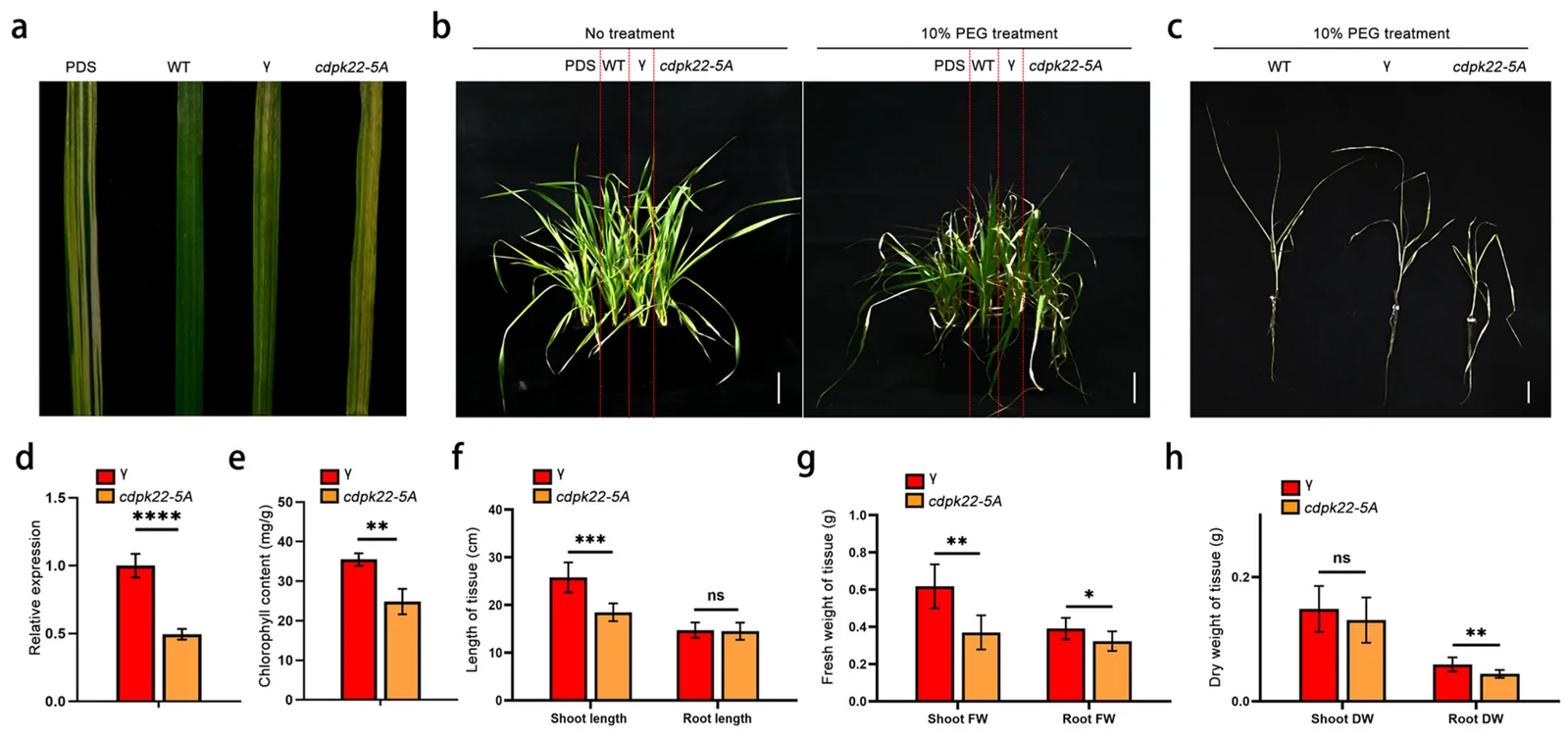
Functional characterization of *TaCDPK22-5A* in wheat drought response using VIGS. (**a**) Validation of the effectiveness of the BSMV inoculation system. (**b**,**c**) Phenotypic observation of *TaCDPK22-5A*-silenced plants derived from the wheat line 23B1 before and after 10% PEG6000 treatment. Scale bar = 6 cm. (**d**) Relative gene expression analysis of *TaCDPK22-5A* in silenced plants. (**e**–**h**) Physiological characterization, including chlorophyll content, shoot and root length, fresh weight, and dry weight of seedling tissues after 10% PEG6000 treatment. PDS represent positive control for VIGS efficiency (BSMV-*PDS*), WT represent seedlings treated with FES buffer, γ represent negative control by empty-vector BSMV-γ, *cdpk22-5A* represent *TaCDPK22-5A* gene-silenced seedlings. Error bars represent the standard deviation (SD) of three biological replicates. Significant differences were determined using multiple unpaired *t* tests (* *p* < 0.05, ** *p* < 0.01, *** *p* < 0.001, **** *p* < 0.0001; ns, not significant).

To further investigate whether the drought-responsive *CDPK* genes identified from transcriptome analysis contribute directly to drought tolerance, virus-induced gene silencing (VIGS) was performed to suppress the expression of *TaCDPK22-5A*. Wheat plants treated with FES buffer alone were used as the wild-type control (WT), while plants inoculated with the empty BSMV-γ vector were used as the negative control. The BSMV-*PDS* construct was used as a positive control to evaluate the efficiency of the VIGS system. Approximately 10 days after inoculation, typical leaf photobleaching symptoms were observed in BSMV-*PDS*-treated plants, confirming the successful establishment of the silencing system (Figure 6a). After confirming the effectiveness of VIGS, the transcript levels of *TaCDPK22-5A* were examined by qRT-PCR. Compared with the BSMV-γ control plants, the expression levels of the target genes were significantly reduced in the corresponding BSMV-*TaCDPK22-5A* seedlings, confirming effective suppression of the target transcript (Figure 6b). These results demonstrated that the VIGS system was suitable for functional analysis of *CDPK* genes in wheat (Supplementary Table S4).

To determine the contribution of *TaCDPK22-5A* to drought tolerance, the silenced plants and control plants derived from the drought-tolerant line 23B1 were subjected to PEG6000-induced drought stress. Under control conditions, BSMV-*TaCDPK22-5A* and BSMV-γ plants showed generally comparable growth phenotypes, indicating that *TaCDPK22-5A* silencing did not cause a pronounced growth defect under non-stress conditions. However, after 10 days of 10% PEG6000 treatment, *TaCDPK22-5A*-silenced plants exhibited more severe wilting symptoms, leaf yellowing, reduced growth compared with the controls (Figure 6a). Physiological measurements further supported the positive role of *TaCDPK22-5A* in drought adaptation. Under PEG6000 treatment, *TaCDPK22-5A*-silenced plants displayed significant reductions in shoot length, fresh biomass and chlorophyll content compared with control plants (Figure 6b). These results suggest that suppression of *TaCDPK22-5A* compromises plant growth maintenance and photosynthetic capacity under drought stress, indicating that *TaCDPK22-5A* may represent a positive regulator associated with drought adaptation in wheat.

## 4. Discussion

Drought stress is a major factor limiting wheat growth and yield stability, and improving drought tolerance requires a deeper understanding of the physiological and molecular processes involved in stress adaptation. In this study, we integrated physiological measurements, transcriptome analysis, qRT-PCR validation, and virus-induced gene silencing (VIGS) to investigate the regulatory mechanisms associated with contrasting drought responses between two wheat lines, 23B1 and 23B39. Under PEG-induced drought stress, 23B1 showed less growth inhibition, greater accumulation of osmoprotective compounds, enhanced antioxidant activity, and reduced membrane damage compared with 23B39, suggesting a stronger capacity to maintain cellular homeostasis under water-deficit conditions. Transcriptome analysis revealed extensive differences in drought-responsive gene expression between the two lines, with enrichment of pathways involved in metabolic regulation, transport processes, secondary metabolism, and stress-associated responses. Among the identified *CDPK* genes, *TaCDPK22-5A* displayed distinct expression patterns between the two wheat lines with contrasting drought responses. Importantly, functional validation using BSMV-mediated gene silencing demonstrated that *TaCDPK22-5A* positively contributes to drought tolerance. These findings extend previous studies on plant CDPK functions and highlight the contribution of genotype-dependent regulation of calcium-associated signaling pathways to wheat drought adaptation.

### 4.1. Physiological Adaptation Under Drought: Enhanced Osmotic Adjustment and Membrane Protection

PEG-mediated osmotic stress is widely used as a reliable approach for simulating drought conditions in controlled environments because it avoids the heterogeneity and secondary effects associated with soil-based assays [25]. In this study, PEG treatment significantly inhibited shoot and root growth in both wheat lines; however, the degree of inhibition differed between 23B1 and 23B39. Although 23B1 exhibited a greater plant height under non-stress conditions, it maintained longer shoots and roots than 23B39 under both moderate (10% PEG) and severe (20% PEG) stress, suggesting a stronger capacity to maintain growth under water-deficit conditions. Drought stress disrupts cellular water balance and promotes excessive production of ROS, which can cause oxidative damage to membrane lipids and proteins [36]. Plants alleviate these effects through multiple physiological adjustments, including osmolyte accumulation and activation of antioxidant defense systems. Compatible solutes such as proline and soluble sugars contribute to osmotic regulation, stabilization of cellular structures, and protection of macromolecules during dehydration [9]. In addition, antioxidant enzymes, including POD, play essential roles in controlling ROS accumulation and maintaining redox homeostasis under drought conditions [37].

In the present study, drought stress induced the accumulation of proline and soluble sugars in both wheat lines, but the magnitude of these responses differed substantially. Compared with 23B39, 23B1 accumulated higher levels of proline and soluble sugars under PEG treatment, particularly under 20% PEG stress. This enhanced osmotic adjustment capacity was accompanied by lower MDA accumulation, indicating reduced membrane lipid peroxidation. Furthermore, POD activity increased progressively in 23B1 under drought stress and was significantly higher than that in 23B39 at both stress levels, suggesting a more effective ROS-scavenging system in the tolerant line. These results indicate that the stronger drought performance observed in 23B1 is associated with coordinated regulation of osmotic protection and antioxidant defense.

Similar physiological patterns have been reported in drought-tolerant wheat genotypes, which often maintain higher osmolyte concentrations and stronger antioxidant activity while limiting oxidative damage compared with susceptible genotypes [8]. Therefore, the contrasting physiological responses observed between 23B1 and 23B39 provide a foundation for further investigation of the molecular mechanisms underlying their different drought adaptation strategies.

### 4.2. Transcriptome Reprogramming Reveals Distinct Drought Response Strategies

Transcriptome profiling provides an effective approach for identifying molecular networks involved in drought adaptation. In this study, RNA-seq analysis showed high reproducibility among biological replicates, with strong correlations within each treatment group, supporting the reliability of the transcriptomic dataset. Comparative analysis revealed extensive transcriptional changes in both wheat lines following PEG treatment, indicating that drought stress triggered broad regulatory responses.

Interestingly, 23B39 exhibited a greater number of DEGs than 23B1 following PEG treatment. A similar pattern has been observed in other crop species, where susceptible genotypes often display more extensive transcriptional changes due to stronger disturbance of cellular homeostasis, whereas tolerant genotypes may activate more selective and efficient regulatory programs [2,38]. In the present study, the larger number of stress-responsive DEGs in 23B39 does not necessarily represent a stronger adaptive response but may reflect greater transcriptional disruption under drought conditions. Functional enrichment analyses further revealed that drought-responsive DEGs were mainly associated with metabolic adjustment, transport regulation, secondary metabolism, and stress-responsive processes. KEGG analysis showed significant enrichment of plant hormone signal transduction and plant–pathogen interaction pathways. These results are consistent with previous studies demonstrating that drought responses are regulated through interconnected networks involving signal perception, metabolic adjustment, hormone regulation, and downstream stress-responsive gene expression [14,39].

Calcium signaling is an important component of the early response to drought-induced changes in cellular water status. Changes in cytosolic Ca^2+^ levels can be decoded by calcium sensors such as CDPKs, which subsequently regulate downstream stress responses through protein phosphorylation [40,41]. In the present study, the enrichment of calcium-related signaling and protein phosphorylation functions, together with the contrasting physiological responses of 23B1 and 23B39, pointed to differential regulation of calcium-dependent signaling as a potential component of their drought response. This prompted us to examine drought-responsive *CDPK* genes and to identify candidates showing consistent differences between the two lines. Importantly, the transcriptome data indicate pathway-level associations rather than direct regulation by individual CDPKs.

### 4.3. CDPKs as Potential Regulators of Drought-Responsive Signaling in Wheat

CDPKs, also known as CPKs, are unique plant-specific serine/threonine kinases that integrate a catalytic kinase domain with a calmodulin-like regulatory domain containing EF-hand Ca^2+^-binding motifs. This structural organization enables CDPKs to directly sense transient changes in intracellular Ca^2+^ concentration and convert them into phosphorylation-mediated signaling events without requiring separate calcium sensor proteins [40,41].

Accumulating evidence indicates that CDPK family members function as pivotal regulators of abiotic stress adaptation. In Arabidopsis, *AtCPK10* contributes to ABA signaling and drought tolerance through phosphorylation of ABF transcription factors [42]. *AtCPK3/4/6/11/27* respond to osmotic stress and activate SnRK2s on multiple phosphosites within the activation loop [43]. *AtCPK6* has been implicated in guard cell signaling and stomatal closure [44], while several rice and maize CDPKs have been shown to regulate ROS homeostasis, osmotic adjustment, and stress-responsive gene expression [45,46,47,48]. In wheat, however, the biological functions of many CDPK family members remain insufficiently characterized despite the availability of whole-genome sequence resources.

By integrating differential expression analysis with transcriptome annotation, we identified 78 CDPK-related DEGs associated with drought treatment. These genes represented the CDPK-related subset of the transcriptomic dataset rather than the complete CDPK family encoded by the wheat genome. The consistency between RNA-seq and qRT-PCR expression patterns of 12 selected candidates strongly supports the reliability of the transcriptomic dataset. More importantly, the distinct expression profiles observed between the tolerant and sensitive lines suggest that differential regulation of specific CDPK members may contribute to the contrasting drought responses observed between the two wheat lines. Although the present transcriptome analysis does not establish a direct regulatory relationship between individual CDPKs and these physiological traits, the concurrent enrichment of calcium-dependent signaling and the contrasting physiological responses support the possibility that differential calcium signaling contributes to the regulation of stress-protective processes in 23B1.

Ta5D02G434800 also exhibited a strong induction pattern in 23B1 but only limited changes in 23B39, indicating that it may represent another CDPK candidate associated with the contrasting drought responses. In the present study, *TaCDPK22-5A* was selected for functional analysis because its expression pattern was consistently associated with drought treatment in 23B1 and was further confirmed by qRT-PCR. This selection does not exclude a potential role of Ta5D02G434800, which remains a candidate for future functional characterization.

### 4.4. Functional Contribution of TaCDPK22-5A to Drought Adaptation in Wheat

Transcriptomic and qRT-PCR analyses identified *TaCDPK22-5A* as a CDPK candidate with contrasting expression patterns between the two wheat lines. To further determine whether these genes contribute directly to drought adaptation, VIGS-mediated gene silencing was performed. The successful reduction of target gene expression in BSMV-*TaCDPK22-5A* plants confirmed the effectiveness of the silencing system and enabled functional evaluation under PEG-induced drought conditions. Silencing of *TaCDPK22-5A* expression significantly impaired the ability of 23B1 plants to maintain growth and physiological activity under PEG-induced drought stress. Although the absence of quantitative measurements under control conditions prevents us from formally testing a genotype × stress interaction, the comparable overall growth observed before PEG treatment and the pronounced phenotypic differences after PEG exposure support a contribution of *TaCDPK22-5A* to plant adaptation to osmotic stress. Together with its drought-induced expression in 23B1, these results support a positive contribution of *TaCDPK22-5A* to drought adaptation, although the downstream physiological processes through which this kinase acts remain to be determined.

The positive role of *TaCDPK22-5A* is consistent with previous studies demonstrating that CDPKs participate in drought signaling by regulating ABA-associated responses, stomatal movement, and downstream stress-responsive genes. Several CDPKs, including *AtCPK6*, *AtCPK10*, and *AtCPK32*, have been shown to regulate ABA-dependent signaling pathways through phosphorylation of transcription factors and ion channels in Arabidopsis [39,49]. Although the downstream targets of *TaCDPK22-5A* remain unknown, its requirement for maintaining drought performance suggests its important role in calcium-associated drought signaling networks in wheat. More direct experimental evidence in wheat is necessary to determine the relationship between *TaCDPK22-5A* and the ABA signaling pathway in drought resistance response.

### 4.5. Limitations and Future Perspectives

Although this study provides integrated physiological, transcriptomic, and functional evidence for the involvement of *CDPK* genes in wheat drought responses, several limitations remain. PEG6000-induced osmotic stress offers a controlled and reproducible system for evaluating drought responses; however, it cannot fully replicate the complexity of field drought conditions, including gradual soil drying, temperature fluctuations, and interactions with environmental microorganisms. Therefore, further evaluation of 23B1 and 23B39 under field drought conditions will be necessary to determine whether the candidate gene contributes to drought performance under natural environmental conditions.

Another potential limitation of comparative analyses using different wheat lines is that phenotypic and transcriptional differences may reflect not only drought-responsive regulatory genes but also variations in the overall genetic background. Although 23B1 and 23B39 showed contrasting drought responses and provided a useful system for identifying stress-associated genes, the contribution of individual candidate genes should be interpreted with caution. In this study, we attempted to reduce this limitation by selecting two wheat lines derived from related breeding materials and by combining comparative transcriptomics with functional validation, and combining transcriptomic analysis with BSMV-mediated gene silencing. The altered drought phenotypes observed after silencing *TaCDPK22-5A* provide direct functional evidence supporting its contribution to drought-responsive regulation. Although silencing *TaCDPK22-5A* resulted in reduced growth, biomass accumulation, and chlorophyll retention under drought stress, the present VIGS experiment did not directly determine whether this gene affects osmolyte accumulation, membrane lipid peroxidation, or antioxidant enzyme activity. Therefore, the relationship between *TaCDPK22-5A* and the physiological responses characterized in this study should be investigated in future work. Measurements of proline, soluble sugars, MDA, and POD activity, together with analyses of ABA accumulation and downstream signaling components, would help clarify how *TaCDPK22-5A* contributes to drought adaptation.

Moreover, the downstream regulatory mechanisms of *TaCDPK22-5A* and the direct regulatory role of this gene in ABA signaling remain largely unknown. As CDPKs function mainly through phosphorylation of downstream substrates, future phosphoproteomic analyses, kinase activity assays, and protein–protein interaction studies will be important for identifying their direct targets and clarifying how they regulate ABA-related drought signaling, oxidative stress responses, and drought-associated physiological processes.

The hexaploid wheat genome contains multiple homoeologous gene copies that may exhibit functional redundancy, subfunctionalization, or distinct expression patterns. Although the present study identified a promising CDPK candidate, a further limitation concerns the potential co-silencing of homoeologous *CDPK* genes during BSMV-mediated VIGS. The *TaCDPK22-5A* silencing fragment was selected based on sequence comparison among the A-, B-, and D-genome homoeologs and gene-specific primers were used for transcript quantification refer to former reports [50,51], but the expression of the corresponding B- and D-genome copies was not directly examined after VIGS. Therefore, partial co-silencing of closely related homoeologs cannot be completely excluded. Future experiments using homoeolog-specific expression analysis and complementary genetic approaches will be required to resolve the individual contribution of *TaCDPK22-5A*.

Finally, a limitation of the VIGS experiment is that quantitative phenotyping was performed primarily under PEG-induced stress. Although representative images indicated no obvious growth differences between the empty-vector and *TaCDPK22-5A*-silenced plants before PEG treatment, quantitative measurements under both control and stress conditions were not available. Therefore, a formal genotype × stress interaction could not be tested. Future experiments using a factorial design with quantitative phenotyping under both control and drought conditions will help determine the extent to which *TaCDPK22-5A* affects basal growth versus stress-specific adaptation.

This study establishes *TaCDPK22-5A* as functional positive regulators associated with contrasting drought responses and provides a foundation for future mechanistic studies and genetic improvement of drought tolerance in wheat.

## 5. Conclusions

In this study, we integrated physiological analysis, transcriptome profiling, and functional validation to provide insights into differential drought responses between wheat lines 23B1 and 23B39. 23B1 exhibited stronger drought adaptation through improved osmotic adjustment, enhanced antioxidant capacity, and reduced membrane damage compared with the sensitive line 23B39. Transcriptomic analysis revealed distinct regulatory patterns between the two lines and highlighted changes in metabolic processes, transport regulation, and stress-associated signaling networks during drought responses. VIGS-mediated silencing further indicated that reduced *TaCDPK22-5A* expression compromises the ability of 23B1 seedlings to maintain growth, biomass accumulation, and chlorophyll content under PEG-induced osmotic stress. Together, these findings support a role for *TaCDPK22-5A*-associated CDPK signaling in wheat responses to osmotic stress and provide a candidate for further investigation of calcium-dependent stress signaling in wheat. Further validation under soil-drying and field conditions will be required to determine its potential value for drought-resilience breeding.

## Data Availability

The raw data supporting the conclusions of this article are available in the China National Center for Bioinformatics database (https://ngdc.cncb.ac.cn/) under accession number PRJCA072055.

## Supplementary Materials

The following supporting information can be downloaded at https://www.mdpi.com/article/10.3390/genes17090985/s1. Table S1: The primer sequences used for qRT-PCR; Table S2: Number of raw sequencing reads and post-quality control (QC) reads for each sample; Table S3: CDPK-like candidates screened out by RNA-seq; Table S4: The primer sequences and mRNA target sequences used for VIGS of *TaCDPK-5A*.
