# Supplementary material for: Integrative Physiological, Transcriptomic, and Functional Analysis Reveals a Positive Contribution of TaCDPK22-5A to Drought Adaptation in Wheat
Source: Genes (Basel). 2026 Aug 22;17(9):985. doi: 10.3390/genes17090985 (PMC13606330; doi:10.3390/genes17090985)
Supplement: Supplementary file 1 [file genes-17-00985-s001.zip › genes-4492265-supplementary.pdf]

Supplementary Table S1. The primer sequences used for qRT-PCR.

Primer name	Forward primer (5′-3′)	Reverse primer (5′-3′)
qTaActin	CCTCTGCGCCAATCGT	TCAGCCGAGCGGGAAATTGT
qTaCDPK22-5A	GGCTTGCGGATAAACGGTCA	ACTCGTCAGTGTCCAAGGTG
qTaCS5A02G426800	CGTCATGCACAGGGATCTCA	GAAGAAGATGGAGAGCCCGA
qTaCS4A02G187900	ATAACAGCGGCACAATCACG	GCTGCTTCCATGAGTTGCTC
qTaCS4A02G432300	ACCTCAAGCCCGAGAACATC	CTTTGACCAGGCTGGGTGTA
qTaCS5B02G297700	AGGAGGGCTTGCGGATAAAC	TCAGTGTCCAAGGTGCCATT
qTaCS5D02G434800	TCTTCTTCACGCCAGGTCAG	ACGTCAACTTCACGCCCATA
qTaCS4D02G125500	GAGGTGTTCAGGGACATCGT	TGACGCCGATACTCCAGATG
qTaCS2B02G478100	TCTTCAAGCCTGGCCAAATC	TCCATACATCAGCTTCTGGC
qTaCS2D02G229100	TCTGAAGCCGGAGAACTT	AGGAACACCGCTGAGAAG
qTaCS2D02G456400	CAAGCCCGAGAACTTCCT	ACACCAGCGGTCCATACA
qTaCS5B02G428400	CGGGACCTCAAGCCCGAGAA	GCCCAAAATGGCGGCACA
qTaCS5D02G475900	CGAGCTCTTCGACAGGAT	ATTGCCTTCAGAGGAGAAC

Supplementary Table S2. Number of raw sequencing reads and post-quality control (QC) reads for each sample.

Sample	Raw Reads No	Raw Bases(Gb)	Q30(Gb)	GC(%)	N(%)	Q20(%)	Q30(%)
23B1CK_1	71809722	10.84	10.43	55.31	0.03659	99.14	96.19
23B1CK_2	101366532	15.31	14.73	55.31	0.036522	99.16	96.24
23B1CK_3	70963494	10.72	10.30	55.26	0.036559	99.12	96.14
23B1Y6_1	90271078	13.63	13.10	54.91	0.036469	99.11	96.07
23B1_6h_2	90683896	13.69	13.17	54.82	0.036572	99.14	96.2
23B1_6h _3	85845378	12.96	12.47	54.79	0.036415	99.13	96.19
23B1_12h_1	75665688	11.43	11.00	54.04	0.036614	99.15	96.24
23B1_12h_2	75216598	11.36	10.93	54.19	0.036606	99.16	96.21
23B1_12h_3	75316842	11.37	10.93	54.11	0.036389	99.12	96.14
23B39CK_1	82647988	12.48	12.00	55.07	0.036453	99.13	96.14
23B39CK_2	83652320	12.63	12.18	54.95	0.004191	99.21	96.41
23B39CK_3	102298950	15.45	14.88	54.89	0.036546	99.18	96.35
23B39_6h_1	76518204	11.55	11.16	54.82	0.003989	99.26	96.59
23B39_6h_2	100479194	15.17	14.56	55.17	0.036532	99.08	95.97
23B39_6h_3	90231520	13.62	13.09	54.97	0.036507	99.1	96.09
23B39_12h_1	92571098	13.98	13.45	54.08	0.036508	99.13	96.2
23B39_12h_2	88507862	13.36	12.87	54.1	0.036501	99.17	96.29
23B39_12h_3	63930216	9.65	9.35	54.08	0.011588	99.34	96.87

Supplementary Table S3. CDPK-like candidates screened out by RNA-seq.

Gene ID	Gene Name
TraesCS1A02G292200	CDPK16
TraesCS1A02G424200	CDPK15
TraesCS1B02G301600	CDPK16
TraesCS1B02G456700	CDPK15
TraesCS1D02G290700	CDPK16
TraesCS1D02G433400	CDPK15
TraesCS2A02G199700	CDPK20
TraesCS2A02G223100	CDPK19
TraesCS2A02G233900	CDPK34
TraesCS2A02G239900	CDPK18

TraesCS2A02G248200	CDPK17
TraesCS2A02G407200	CDPK12
TraesCS2A02G456100	CDPK13
TraesCS2B02G227000	CDPK20
TraesCS2B02G248700	CDPK19
TraesCS2B02G269200	CDPK17
TraesCS2B02G424900	CDPK12
TraesCS2B02G478100	CDPK13
TraesCS2D02G207400	CDPK20
TraesCS2D02G229100	CDPK19
TraesCS2D02G245700	CDPK18
TraesCS2D02G249900	CDPK17
TraesCS2D02G404200	CDPK12
TraesCS2D02G456400	CDPK13
TraesCS3A02G224900	CDPK1
TraesCS3A02G334200	CDPK2
TraesCS3A02G351300	CDPK3
TraesCS3B02G254500	CDPK1
TraesCS3B02G365200	CDPK2
TraesCS3B02G383800	CDPK3
TraesCS3D02G228700	CDPK1
TraesCS3D02G327700	CDPK34
TraesCS3D02G345500	CDPK3
TraesCS4A02G187900	CDPK17
TraesCS4A02G206200	CDPK24
TraesCS4A02G283400	CDPK9
TraesCS4A02G432300	CDPK17
TraesCS4B02G030100	CDPK9
TraesCS4B02G109800	CDPK24
TraesCS4B02G130300	CDPK17
TraesCS4B02G321800	CDPK7
TraesCS4D02G027600	CDPK9
TraesCS4D02G107200	CDPK24
TraesCS4D02G125500	CDPK25
TraesCS4D02G318400	CDPK7
TraesCS5A02G107900	CDPK28
TraesCS5A02G118200	CDPK29
TraesCS5A02G298500	CDPK22
TraesCS5A02G426500	CDPK10
TraesCS5A02G426800	CDPK11
TraesCS5A02G463100	CDPK8
TraesCS5A02G490200	CDPK7
TraesCS5B02G109300	CDPK27
TraesCS5B02G112900	CDPK29
TraesCS5B02G115400	CDPK28
TraesCS5B02G137600	CDPK25
TraesCS5B02G297700	CDPK22
TraesCS5B02G428400	CDPK10
TraesCS5B02G428700	CDPK11
TraesCS5B02G474500	CDPK8

TraesCS5D02G119100	CDPK29
TraesCS5D02G122800	CDPK28
TraesCS5D02G124000	CDPK27
TraesCS5D02G153800	CDPK25
TraesCS5D02G305200	CDPK22
TraesCS5D02G434500	CDPK10
TraesCS5D02G434800	CDPK11
TraesCS5D02G475900	CDPK8
TraesCS6A02G089400	CDPK4
TraesCS6A02G261200	CDPK5
TraesCS6B02G111800	CDPK4
TraesCS6B02G288600	CDPK5
TraesCS6D02G242000	CDPK5
TraesCS7A02G267000	CDPK21
TraesCS7D02G052900	CDPK17
TraesCS7D02G267700	CDPK21
TraesCSU02G016900	CDPK4
TraesCSU02G091900	CDPK17

Note: The supporting information and data for transcriptomic analysis visualization are available at: https://doi.org/10.5281/zenodo.21476989.

Supplementary Table S4. The primer sequences and mRNA target sequences used for VIGS of *TaCDPK-5A*.

Name	Sequence (5′-3′)	Sequence (3′-5′)
VIGS-primer	AGCTGAGCGGCCGCCCCGGGAC GGTCATGCTGTTCCTGAG	AGCTGATTAATTAACCCGGGTTC CTCCAGTCTGAGCCAGA
Target sequence	ACGGTCATGCTGTTCCTGAG	TCTGGCTCAGACTGGAGGAA
